# Common arboviruses and the kidney: a review

**DOI:** 10.1590/2175-8239-JBN-2023-0168en

**Published:** 2024-07-29

**Authors:** Gabriel Rotsen Fortes Aguiar, Geraldo Bezerra da Silva, Janaína de Almeida Mota Ramalho, Nattachai Srisawat, Elizabeth de Francesco Daher

**Affiliations:** 1 Universidade Federal do Ceará, Faculdade de Medicina, Programa de Pós-Graduação em Ciências Médicas, Departamento de Medicina Interna, Fortaleza, CE, Brazil.; 2 Universidade de Fortaleza, Centro de Ciências da Saúde, Curso de Medicina, Fortaleza, CE, Brazil.; 3 Chulalongkorn University, Faculty of Medicine, Department of Medicine, Division of Nephrology, Center of Excellence for Critical Care Nephrology, and Tropical Medicine Cluster, Bangkok, Tailândia.

**Keywords:** Arboviruses, Dengue, Chikungunya Virus, Zika Virus, Kidney, Prognosis

## Abstract

Arboviruses are endemic in several countries and represent a worrying public health problem. The most important of these diseases is dengue fever, whose numbers continue to rise and have reached millions of annual cases in Brazil since the last decade. Other arboviruses of public health concern are chikungunya and Zika, both of which have caused recent epidemics, and yellow fever, which has also caused epidemic outbreaks in our country. Like most infectious diseases, arboviruses have the potential to affect the kidneys through several mechanisms. These include the direct action of the viruses, systemic inflammation, hemorrhagic phenomena and other complications, in addition to the toxicity of the drugs used in treatment. In this review article, the epidemiological aspects of the main arboviruses in Brazil and other countries where these diseases are endemic, clinical aspects and the main laboratory changes found, including changes in renal function, are addressed. It also describes how arboviruses behave in kidney transplant patients. The pathophysiological mechanisms of kidney injury associated with arboviruses are described and finally the recommended treatment for each disease and recommendations for kidney support in this context are given.

## Chikungunya

Chikungunya (CHIKV) is an *Alphavirus* from the *Togaviridae* family that is transmitted by mosquitoes (*Aedes aegypti* and *Aedes albopictus*). “Chikungunya” means “those who bend”, and is an African expression that refers to the movement that patients with joint pain make. The disease can be mild and cause no complications, but the major problem is the development of chronic arthritis, which can last for several months or even years^1,2,3^.

## History and Epidemiology

Chikungunya causes a significant number of cases each year and frequent outbreaks worldwide^2^. The first report was in 1952, and since then more than a hundred countries have recorded CHIKV infection, from Africa to Asia, from Oceania to the Americas, and recently Europe has also been affected by arboviruses. In 2004, the virus was detected in Kenya and then spread to the Indian Ocean islands, where it caused a severe epidemic^4^.

An important step in establishing transmission was that the virus reached the urban cycle through the two anthropophilic mosquitoes of the genus Aedes, after millions of cases were reported worldwide, until it had fully adapted to this new environment and is now a real threat^7^.

The first report of chikungunya in the Americas was in 2013, during the outbreak in Saint Martin in the Caribbean. This was followed by an epidemic in South America, affecting more than 50 countries and subsequently causing around 1 million infections^8^.

Chikungunya was first detected in Brazil in 2014 in the states of Bahia and Amapá. An epidemiological study indicated an incidence rate, mortality rate, and case fatality rate of 114.70 and 87.59 per 100,000 inhabitants, 0.15 and 0.12 per 100,000, and 0.13% and 0.14% for 2016 and 2017 respectively. Most cases affected young women with mean age 20–49 years, people with brown skin, and residents of urban areas^8^.

Adaptation to new vectors and their spread to temperate climates, associated with disorganized urban sprawl and high human density, has increased the incidence of Chikungunya infection.

Concerns have therefore been raised regarding the occurrence of chikungunya in high-income countries, where outbreaks have been reported in Italy and France in the last decade^5^. Several modeling studies predict that climate change can cause exposure to arbovirosis as vectors spread to other regions outside the tropical zone of the globe, and that it may also lead to a high economic burden since chikungunya can cause debilitating disease^9,10,11^.

## Clinical Manifestations

CHIKV has a tropism for different human cell types. The virus replicates within 8 hours and circulates in the lymphatic vessels and bloodstream, with the possibility of reaching different infection sites. Joints and muscles are important targets for the chikungunya virus in humans. It causes a severe inflammatory reaction affecting various organs, with severe complications in the central and peripheral nervous system, heart, lungs, liver, vascular system and kidneys^12,13^.

The infection is characterized by an acute, post-acute and chronic phase. The clinical presentation can be mild, with few symptoms and no complications, or may develop with severe manifestations, including neurological syndromes and chronic joint disease^14^.

### Acute Disease

The acute stage lasts approximately one week. The incubation period lasts 3 to 7 days. This is usually followed by fever, chills, a maculopapular rash, which usually appears after 3 to 4 days of infection, and joint pain. At the same time as the peak of viremia, the fever is high (>39°C) and lasts for more than a week. Defervescence may occur after 4–5 days_1,6,15,16_.

The debilitating joint pain starts in 2–5 days after the fever, and manifests as arthritis or polyarthralgia, which is usually bilateral and symmetrical, preferentially affecting the hands, wrists, shoulders, knees, ankles, and feet. The majority of patients recover, but chronic joint pain can occur, leading to severe disability^6^.

Although rare, neurological manifestations, ranging from encephalopathy to acute disseminated encephalomyelitis, are worrying symptoms that can occur in the acute phase. Guillain-Barré syndrome was described in the 2006 outbreak on Reunion Island^17,18^.

A chikungunya infection usually shows a high viremia, which is higher than with the other arboviruses. Thrombocytopenia is less pronounced than in dengue, and hemorrhagic complications are rare. Lymphopenia is the most common laboratory finding, with lymphocyte counts below 1,000 cells/mm^3^. A mild increase in hepatic enzymes can occur. Hypocalcemia has been reported, the mechanisms of which are not precisely known^1,6,16^.

### Post-Acute and Chronic Phase

The chronic phase can last several months and even years^1,3^. The post-acute phase, in which symptoms persist after 14 days, is observed in 75% of cases. Approximately 30 to 40% of patients develop chronic arthritis6. The chronic form of chikungunya infection is considered when the clinical manifestations persist for more than 3 months. Age >40 years, female sex, and increased viral load during the acute phase are considered risk factors for chronic joint disease in chikungunya^6,19^.

There is evidence of a direct viral effect in chikungunya-associated joint disease, once viral genetic material is found in synovial biopsies, and synovial macrophages are potential reservoirs for CHIKV^14^.

The pathophysiology of chronic chikungunya infection is not completely understood. Immunological phenomena are thought to be more relevant than the direct effects of the virus, which occur in CHIKV patients in the form of an abnormal immune response. Those with chronic joint disease have higher IL-6 levels, as well as IL-7 and an abnormal RANKL to osteoprotegerin ratio, which is associated with bone disease and then contributes to the joint disease observed in chikungunya^6^.

## Kidney Involvement

Kidney complications in chikungunya varies and needs to be better investigated.

### Pathology

The most common histopathological finding in chikungunya-associated kidney injury is acute interstitial nephritis associated with acute tubular necrosis, mononuclear infiltrate, glomerular congestion, and nephrosclerosis. In the acute phase of the disease, viral antigens have been detected by immunofluorescence in kidney tissue, specifically in tubular cells, but there is no evidence of persistence in the chronic phase. In a study of 5 cases with CHIKV-associated kidney lesions, two predominant types were found in kidney biopsies: acute interstitial nephritis and acute tubular injury^20^.

A study in Brazil analyzing patients with biopsy-proven kidney injury established that the most frequent diagnoses were focal and segmental glomerulosclerosis and most were associated with autoimmune phenomena, suggesting that chikungunya is a trigger for auto-immunity^21^.

A study analyzing 13 fatal chikungunya cases from Colombia found that 11 cases had acute interstitial nephritis, 10 cases had congestion and glomerular edema, 5 cases had acute tubular necrosis, 5 cases had nephrosclerosis, and 1 case had membranoproliferative glomerulonephritis^22^.

### Kidney Disease in Chikungunya

Kidney disease in chikungunya ranges from 21 to 45%^23^. Severe cases of chikungunya can lead to acute kidney injury (AKI) in a frequency as high as 79%, and are associated with higher mortality^23^. One case report described a male patient with fever, myalgia, and anuria^25^. On investigation, AKI was detected, and rhabdomyolysis was suspected as the cause, induced by chikungunya^25^.

A study analyzing deaths from Puerto Rico in 2014 identified thirty CHIKV-infected fatal cases. Among the histopathological findings of the 11 samples in which the viral antigen was detected in the kidney, the study described extensive glomerulosclerosis in 3 cases, mild/moderate glomerulosclerosis in 5 cases, atherosclerosis in 7 cases, an interstitial infiltrate of mononuclear cells in 10 cases, and interstitial fibrosis in 7 cases. CHIKV was detected in the glomeruli in 4 patients, in the interstitial connective tissue/capsule in 2 patients, and in the tubular epithelium in one patient^26^. Other studies in which kidney samples from CHIKV patients were examined demonstrated interstitial nephritis and tubular injury with non-necrotizing epithelioid cells, giant cell granulomas, focal segmental glomerulosclerosis and thrombotic microangiopathy^27,28^.

Immunological mechanisms are involved in the process of CHIKV kidney disease. There is an imbalance in the immune response with evidence of IgM-type antibody production and the formation of autoantibodies, including cryoglobulins, which have kidney-damaging properties^23^. The virus can escape the immune system, and there may also be an interaction between genes and the environment that determines each patient’s response to the infection^23^. Kidney tissue could also serve as a CHIKV reservoir, as this virus has the ability to persist in various organs and tissues^23^.

There is evidence that chikungunya is a trigger for various kidney diseases, including glomerulonephritis^23^. In a study of 15 chikungunya patients in Brazil who underwent kidney biopsy, the following histopathological lesions were identified: focal segmental glomerulosclerosis (FSGS), class IV lupus nephritis, crescentic glomerulonephritis, thrombotic microangiopathy, pauci-immune vasculitis, PLA2R-positive membranous nephropathy, and collapsing glomerulosclerosis^23^.

**Figure 1:**
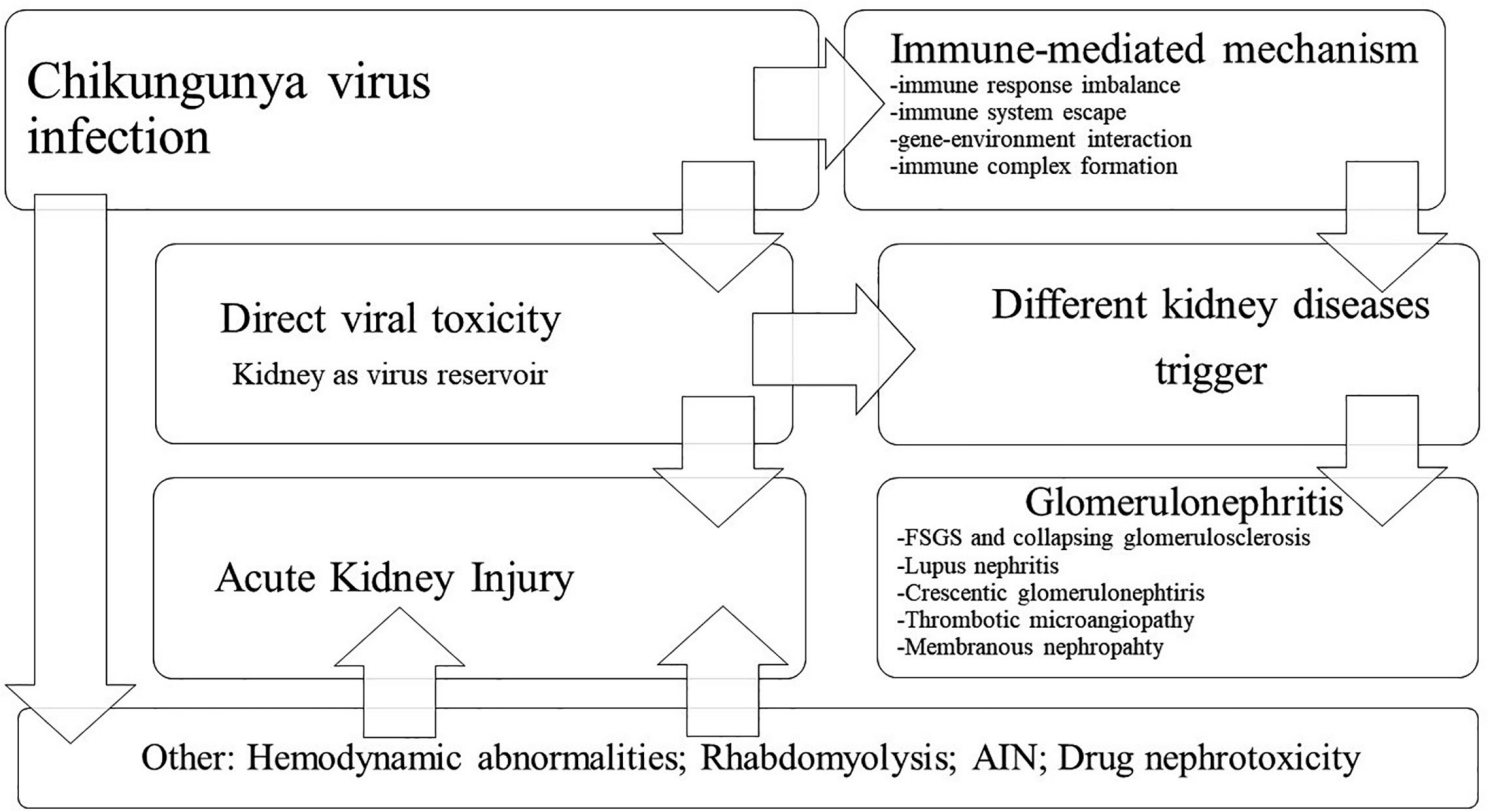
Pathophysiology of kidney involvement in Chikungunya virus infection. TMA: Thrombotic Microangiopathy; AIN: Acute Interstitial Nephritis.

The pathophysiology of kidney involvement in chikungunya virus infection is shown in Figure 1.

### Laboratorial Findings

Proteinuria and hematuria have been found in CHIKV and are more frequent among those with arthritis^23^. Proteinuria is found in 10 to 20% of cases, and hematuria in less than 5% of cases. Creatinine increase, which classifies patients with AKI or chronic kidney disease (CKD), is a relatively frequent complication of CHIKV and is described in 5.2% to 79% of cases^23^.

### Other Kidney Manifestations

Nephritic syndrome has been described in association with chikungunya in a young man from India who had no history of kidney disease. Genetic material from CHIKV was detected in the serum of the patient^29^.

## Treatment

Chikungunya treatment is essentially for pain relief in the absence of a specific drug^31^, and treatment remains symptomatic. For some specific glomerulonephritis triggered by chikungunya, such as FGSF or thrombotic microangiopathy, there are specific treatments with corticosteroids, immunosuppressants, plasmapheresis and others, but there is insufficient evidence for any of these treatments in chikungunya^31^.

## Zika

The Zika virus (ZIKV) is a *Flavivirus* from the *Flaviviridae* family that was first identified in 1947 in the region of the forest of the same name in Uganda. The first human infection was described a few years later in Nigeria, and for many years only a few cases were documented^32^. Transmission also occurs through the bite of the *Aedes* mosquito, and human-to-human transmission has been described in addition to perinatal, sexual, and breast milk transmission.

## History and Epidemiology

Until 2007, Zika was only responsible for small outbreaks, when a major epidemic occurred on the Pacific island of Yap, affecting around 75% of the population. Local physicians initially described a “dengue-like illness”, but some patients, who only reported subjective fever and conjunctivitis, raised suspicions. This epidemic was followed by a larger epidemic in French Polynesia in 2013-2014 with more than 30,000 cases^33^. In 2015, ZIKV was detected in the Americas. Since then, 33 countries have reported autochthonous transmission and a significant increase in some complications, including microcephaly and Guillain-Barré syndrome^33^. ZIKV has also been detected in Europe, suggesting rapid spread to other parts of the world, similar to chikungunya and dengue^32,33^.

## Clinical Manifestations

ZIKV has an incubation period of 3–14 days. Initial symptoms are similar to those of influenza, with fever and malaise. Zika is characterized by a maculopapular rash with itching and joint pain. Although rare, ZIKV can cause Guillain-Barré syndrome and other neurological complications in adults^33,34,35^.

Recent outbreaks have raised worrying questions about the potential severity of ZIKV, mainly regarding its association with neonatal complications^38^. In Brazil, a study of pregnant women infected with ZIKV showed that 42% of fetuses had ultrasound abnormalities^39^. Congenital Zika disease has a variable presentation, ranging from fetal death to microcephaly and other manifestations^33^.

The occurrence of ZIKV has become a public health concern due to its impact on neonatal health and neurological complications, particularly Guillain-Barré syndrome in adults, during outbreaks^32^. Zika-associated neuropathy is thought to be caused by antibodies produced against the ZIKV that reacts to peripheral nerves and other structures^33^.

## Kidney Involvement

Kidney involvement in Zika is not well studied. There is some evidence of possible mechanisms causing Zika-associated kidney disease, including persistent excretion of ZIKV in the urine, high susceptibility of glomerular and tubular cells to ZIKV infection, and release of cytokines associated with inflammation^40^. ZIKV is able to invade and replicate in glomerular cells, and the susceptibility of these cells to the virus contributes to its persistence in the urine of infected patients^43^.

An experimental study with different cell types (microglia, fibroblasts, embryonic kidney, and macrophages) inoculated with ZIKV indicated that the human embryonic kidney cell line is a suitable environment for ZIKV replication^44^. A recent study investigated whether ZIKV infection in kidney cells is dependent on glucose levels and demonstrated that glucose levels influence ZIKV replication and have an impact on kidney cell survival^40^. Experimental studies also demonstrated that ZIKV RNA was detected in both glomerular and tubular cells. CD8+ cells were found to be increased among ZIKV-infected kidneys^45^. In a case series of 5 babies who died of ZIKV infection, no viral antigens were found in the kidneys^46^.

It is known from experimental models of Zika virus infection that the kidney is damaged, with significant tubular injury, tubulointerstitial fibrosis (14 days post-infection) and infiltration of immune cells^47^. In this animal model of Zika-associated AKI, non-traditional biomarkers of kidney injury have been detected, such as kidney injury molecular-1 (KIM-1) and neutrophil gelatinase-associated lipocalin (NGAL)^47^. There is evidence that Zika virus induces inflammation and kidney injury through mechanisms involving the Nod-like receptor 3 (NLRP3) inflammasome and secretion of interleukin-1β (IL-1β)^47^.

**Figure 2:**
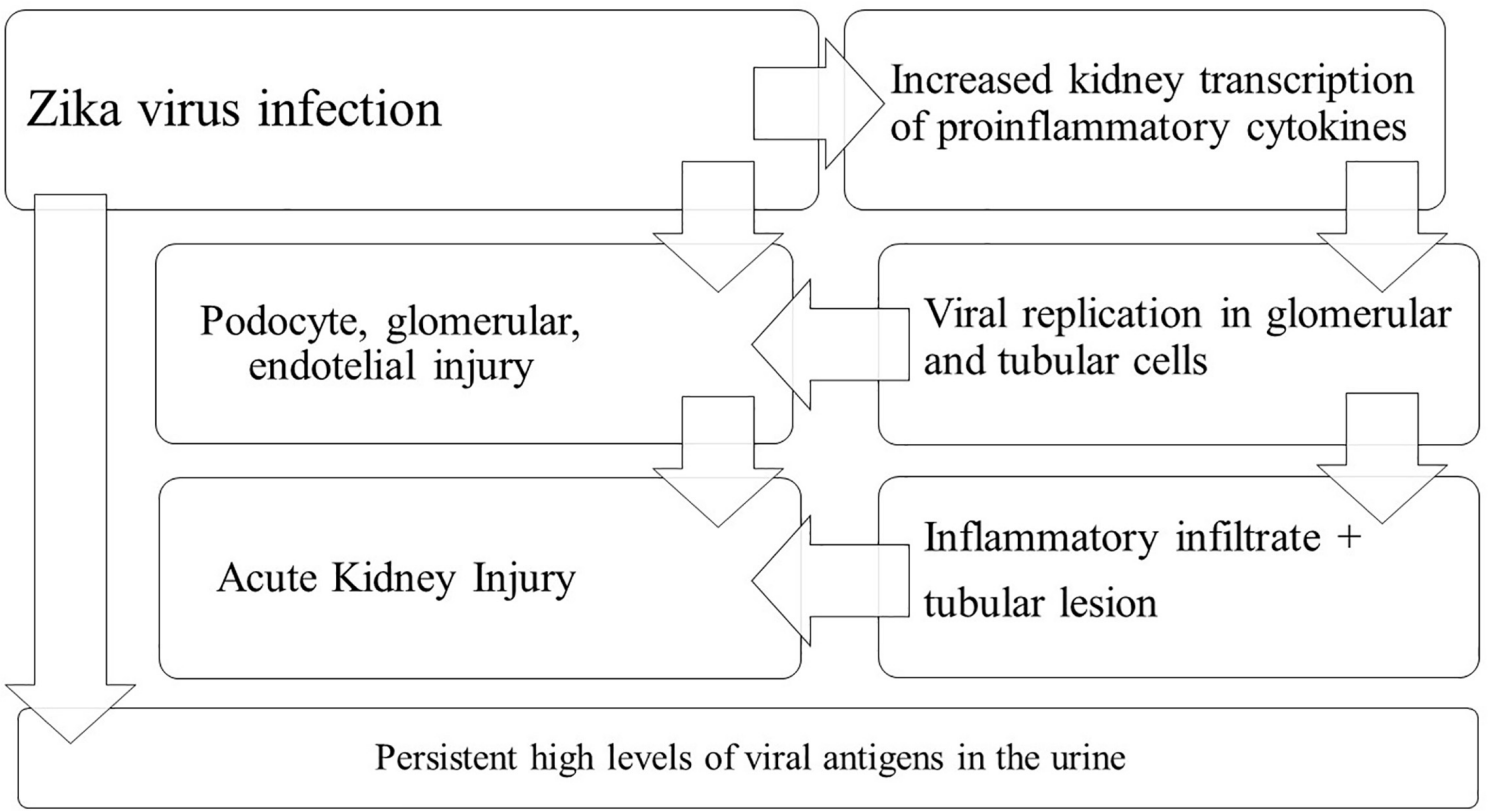
Pathophysiology of kidney involvement in Zika virus infection.

Figure 2 shows an illustration of Zika-associated kidney involvement.

## Treatment

Similar to other arboviruses, there is no specific treatment for Zika, no antiviral or other drug that is effective. Treatment is limited to symptom relief^33^. There are no specific treatment recommendations for Zika-induced AKI. We should follow the current guidelines for AKI treatment.

## Dengue

Dengue is a *Flaviviridae* virus with 4 serotypes (DENV 1-4) transmitted by *Aedes* mosquitoes^48^. The disease can manifest itself from an asymptomatic infection to a severe disease with multi-organ failure. Dengue is currently a major public health problem due to its widespread distribution in the world and the large number of people exposed to the risk of infection^48^.

## History and Epidemiology

The first records of dengue date back to Asia in ancient times, and by the 18th century various epidemics of the dengue-like disease had been recorded in Asia and the Americas^49^. In the 19th and 20th centuries, the virus possibly spread throughout the tropical world^49^.

Dengue is now considered a hyperendemic disease in many parts of the world, with cases reported in more than 100 countries. In the last 50 years, the number of cases has increased 30-fold^48,50^. The World Health Organization (WHO) has an objective to reduce dengue preventable deaths to zero by 2030. In order to achieve this goal, joint measures should be taken^51,52^.

## Clinical Manifestations

Most cases of dengue are asymptomatic. After an incubation period of around 4–8 days, symptoms suddenly appear, including fever, headache, retro-orbital pain, myalgia and other less specific symptoms. In most cases, recovery occurs within a few days, and a small proportion of patients evolve with complications, mainly due to plasma leakage, with or without hemorrhagic manifestations^48,49^. The disease can follow three phases: fever, critical phase and recovery. In the acute phase, the manifestations resemble those of other viral diseases, and this phase can last from 3 to 7 days. Laboratory tests can show leukopenia, thrombocytopenia and hemoconcentration associated with an increase in hepatic enzymes^48,49^.

There is a critical phase that coincides with the decline of fever in which capillary permeability increases and can lead to hypovolemia and organ failure. An abnormal immune response is thought to cause a cytokine storm with the release of several soluble factors, including TNF-α, IL-6, IL-8, IL-10, and IL-12, leading to an increase in vascular permeability and plasma leakage. During the recovery phase, this vascular leakage should end with fluid reabsorption^48,50^.

In 2009, the WHO has proposed a new classification for dengue based on the presence of warning signs, replacing the old classification of dengue fever × dengue hemorrhagic fever^48,50,53^.

## Kidney Involvement

### Pathology

Case studies in adults and children with dengue who underwent kidney biopsies revealed the following findings: glomerular congestion, plasma cell infiltrate, glomerular and peritubular capillary congestion, hyaline casts, endothelial edema, and mesangial proliferation^54,55^.

In dengue-infected patients, deposits of IgG, IgM, and C3 are found in the glomeruli in approximately 50% of cases^57^.

Involvement of the tubular system has also been investigated, with tubular epithelial denudation, tubular cells with pyknotic nuclei, focal tubular atrophy/fibrosis and tubulointerstitial nephritis^54,55^.

In a case series from India, biopsies from 3 dengue-infected patients were analyzed and proximal tubular necrosis with hemorrhage into Bowman’s space was noted in all cases and erythrocyte casts in two cases^58^.

IgA deposits were detected in one case report, suggesting IgA nephropathy, but this association is still not well explored^59^.

### Acute Kidney Injury

The mechanism of dengue-associated AKI is complex. Kidney tubular injury may be associated with both hemodynamic abnormalities and direct viral effects. Other factors such as cytokine-induced injury, hemolysis and rhabdomyolysis play an important role in dengue AKI^50,60^.

In Thailand, a parallel classification of dengue fever has been proposed that categorizes the severity of the disease into dengue fever, dengue hemorrhagic fever, and dengue shock syndrome to capture the occurrence of AKI through the development of the disease^57^. The authors analyzed the medical records of 1,484 dengue patients from a local pathology center and found an AKI prevalence of 4.8%. They concluded that age, male sex, diabetes, obesity, severe dengue fever, and secondary bacterial infection were significantly associated with AKI development^60^.

**Figure 3:**
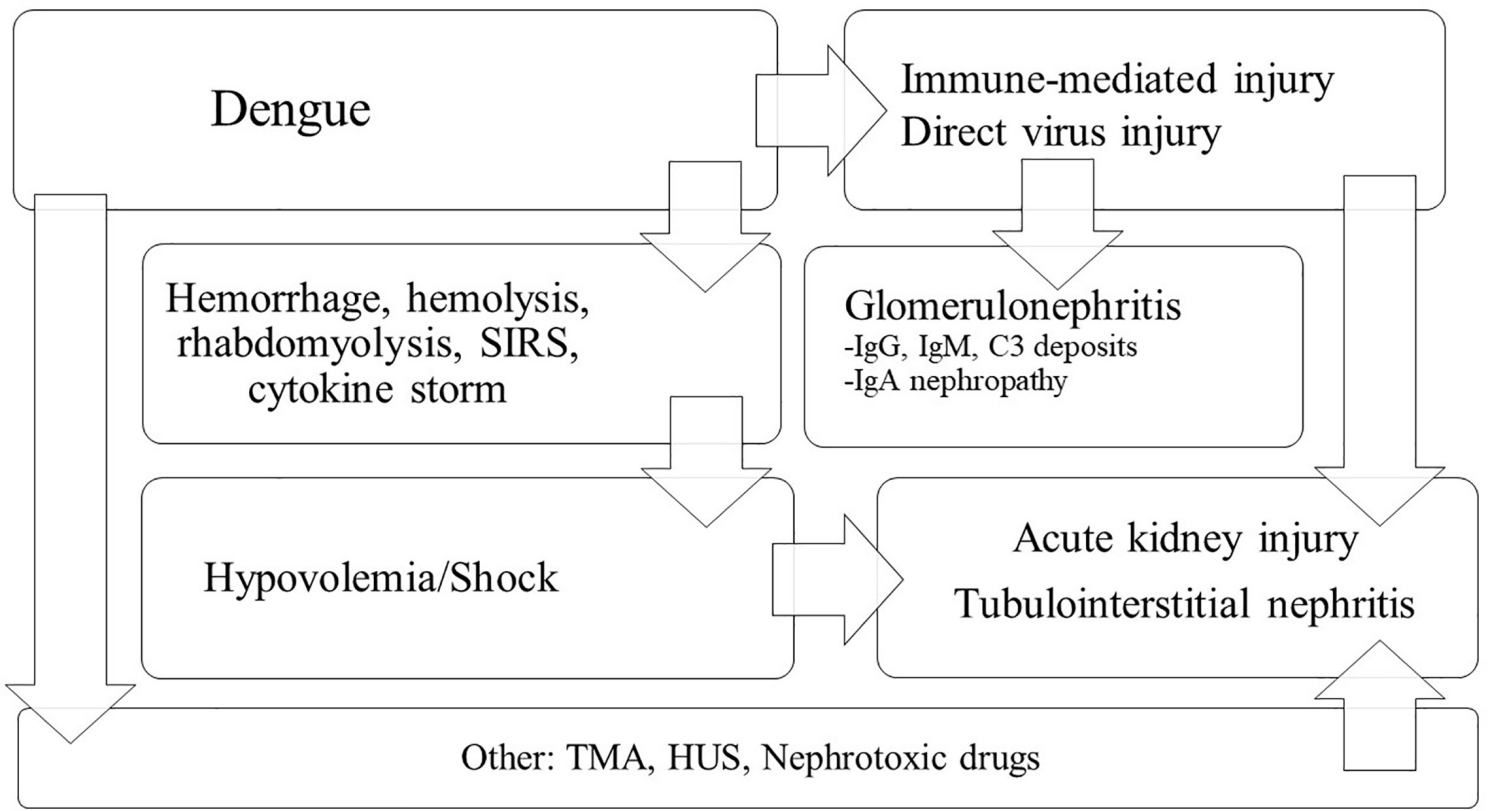
Pathophysiology of kidney involvement in Dengue. SIRS: Systemic inflammatory response syndrome; TMA: Thrombotic Microangiopathy; HUS: Hemolytic-uremic syndrome.

The pathophysiology of kidney involvement in dengue fever is shown in Figure 3.

### Laboratorial Findings

Hematuria has been reported in up to 12.5% of patients with DHF11. Proteinuria is observed in more than 7% of dengue cases^61^ reported two patients with dengue and nephrotic proteinuria^61^. A study performed in Thailand demonstrated urinary sediment abnormalities in dengue patients with AKI, including proteinuria (70.4%), hematuria (59.3%), and pyuria (40.7%). Metabolic acidosis was also found, in 24.3% of patients^59^.

### Cytokines

The most frequent involved cytokines are IL-17, IL-18 and TNF-α. Autoimmune disorders have been associated with dengue, and patients with this infection are now considered to be at high risk of developing several diseases, such as arthritis, multiple sclerosis, vasculitis, lupus and other autoimmune diseases^62^.

### MicroRNAs

Small non-coding RNAs, so-called microRNAs (miRNAs), which have a length of 20–22 nucleotides, are important for the post-transcriptional control of gene expression. Patients with severe dengue present a different pattern of miRNA expression, and there are miRNAs that could be used as prognostic markers for dengue severity, the most promising being miR-574-5p and miR-1246^63^.

### Cortisol

There is evidence of cortisol changes in dengue. Patients with severe dengue seem to have increased levels of cortisol, but the exact meaning of this remains unclear^64^.

## Treatment

Supportive therapy along with fluid administration is the mainstay treatment for dengue. There is an ongoing debate about the use of parenteral corticosteroids in severe dengue^65^. There is a WHO guideline for dengue management^64^. Patients should be cautioned against taking anti-inflammatory drugs because of the risk of bleeding in severe thrombocytopenia^50,67,68^.

There is no specific treatment for AKI associated with dengue, but a crucial step for patient management is fluid support, maintaining adequate perfusion^69^. Fluid intake should be carefully monitored to avoid fluid overload, as intravascular extravasation in dengue is an eminent risk and worsens prognosis. One factor contributing to AKI in dengue is rhabdomyolysis, although its actual incidence is not precisely known. However, this is another reason why fluid administration in dengue is important and essential to prevent AKI or at least decrease its severity. Electrolyte disturbances are also a concern in dengue, and the most frequent is hyponatremia. Therefore, volume replacement should have higher tonicity in these cases^69^.

Severe AKI associated with dengue may require dialysis, and this is often indicated when patients have uremia, hypercatabolism, metabolic acidosis, hyperkalemia, and hypervolemia refractory to clinical measurements^69^. Dengue can cause hemodynamic instability, so continuous hemodialysis has been indicated in patients with dengue-associated AKI in this context^69^.

## Kidney Transplant

Arbovirus infections are also a concern for kidney transplant patients. Particular aspects of chikungunya, Zika, and dengue deserve to be highlighted in the context of kidney transplantation.

Chikungunya infection has been reported to occur more frequently in immunocompromised patients^70^. There are few studies on chikungunya in kidney transplant patients with no complications and low mortality^71^. Also, during a chikungunya infection, the kidney graft may lose function, but fully recovers after the acute episode^71^.

Zika can be transmitted by transfusions and transplantation^70^. Zika infections in kidney transplanted patients are not frequently reported, but when it occurs, the infection seems to be worse in transplant recipients, with loss of allograft function but low mortality^70^. Of concern is that Zika virus can persist in organs even in the absence of viremia, but it is unclear what the risk of complications is^70^.

Dengue virus infection can cause a prolonged viremia and asymptomatic disease, making transmission through kidney transplantation a real risk^70^. Despite this potential risk, serologic testing for dengue virus is not routinely performed in kidney donors or recipients. The incidence of dengue infection does not appear to be higher in the post-transplant period. The reason for this is that most patients have already been infected before transplantation, and the virus seem to be less pathogenic when transmitted by blood transfusion or organ transplantation^70^.

Regarding clinical manifestations, dengue in kidney transplant patients is mild in most cases^70^. There are few reports of severe complications associated with dengue in kidney transplant recipients, including death, and some authors recommend screening for dengue in the preoperative period^73^. Previous studies on kidney transplant-associated dengue show a clinical course not different from that observed in the immunocompetent host, with a low mortality rate^74^. Some symptoms such as fever, myalgia, arthralgia, and headache are even less frequent in kidney transplant patients with dengue than in the general population, but a higher mortality rate has been observed in some series (8.9% versus 3.7% in non-transplant patients)^75^. Immunosuppression is possibly associated with patients’ outcome. High doses of corticosteroids (>7.5 mg per day) are associated with more severe disease. Tacrolimus has been associated with a higher risk of bleeding, and cyclosporine with milder disease^70^.

## Conclusion

Arboviruses are endemic in most tropical countries and can cause kidney injury. There are three main arboviruses, namely chikungunya, Zika, and dengue, which have periodic outbreaks in many parts of the world. There are different mechanisms through which these viruses can cause kidney damage, including the direct effect of the virus. Kidney transplant recipients may also be affected; although damage to the transplant may occur, kidney function generally recovers after the infection subsides. In endemic areas, continuous monitoring must be carried out, along with monitoring of kidney function in all infected patients, including kidney transplant recipients in endemic areas. No specific treatment is available for these viral infections, and supportive therapy, including dialysis for severe kidney injury, remains the mainstay of treatment.

## References

[1] Lima Cavalcanti TYV, Pereira MR, Paula SO, Franca RFO (2022). A review on chikungunya virus epidemiology, pathogenesis and current vaccine development. Viruses.

[2] Costa LB, Barreto FKA, Barreto MCA, Santos THP, Andrade MMO, Farias LABG (2023). Epidemiology and economic burden of chikungunya: a systematic literature review. Trop Med Infect Dis.

[3] Constant LEC, Rajsfus BF, Carneiro PH, Sisnande T, Mohana-Borges R, Allonso D (2021). Overview on chikungunya virus infection: from epidemiology to state-of-the-art experimental models. Front Microbiol.

[4] Caglioti C, Lalle E, Castilletti C, Carletti F, Capobianchi MR, Bordi L (2013). Chikungunya virus infection: an overview. New Microbiol.

[5] Bettis AA, Jackson MLA, Yoon IK, Breugelmans JG, Goios A, Gubler DJ (2022). The global epidemiology of chikungunya from 1999 to 2020: a systematic literature review to inform the development and introduction of vaccines. PLoS Negl Trop Dis.

[6] Bartholomeeusen K, Daniel M, LaBeaud DA, Gasque P, Peeling RW, Stephenson KE (2023). Chikungunya fever. Nat Rev Dis Primers.

[7] Gossner CM, Fournet N, Dias JG, Martínez BF, Del Manso M, Young JJ (2020). Risks related to chikungunya infections among european union travelers, 2012-2018. Emerg Infect Dis.

[8] Vidal ERN, Frutuoso LCV, Duarte EC, Peixoto HM (2022). Epidemiological burden of Chikungunya fever in Brazil, 2016 and 2017. Trop Med Int Health.

[9] Cardona-Ospina JA, Henao-SanMartin V, Paniz-Mondolfi AE, Rodríguez-Morales AJ (2015). Mortality and fatality due to Chikungunya virus infection in Colombia. J Clin Virol.

[10] Cardona-Ospina JA, Diaz-Quijano FA, Rodríguez-Morales AJ (2015). Burden of chikungunya in Latin American countries: estimates of disability-adjusted life-years (DALY) lost in the 2014 epidemic. Int J Infect Dis.

[11] Rezza G, Nicoletti L, Angelini R, Romi R, Finarelli AC, Panning M, CHIKV Study Group (2007). Infection with chikungunya virus in Italy: an outbreak in a temperate region. Lancet.

[12] Khongwichit S, Chansaenroj J, Chirathaworn C, Poovorawan Y (2021). Chikungunya virus infection: molecular biology, clinical characteristics, and epidemiology in Asian countries. J Biomed Sci.

[13] Lima Cavalcanti TYV, Pereira MR, de Paula SO, Franca RFO (2022). A review on Chikungunya virus epidemiology, pathogenesis and current vaccine development. Viruses.

[14] Silva LA, Dermody TS (2017). Chikungunya virus: epidemiology, replication, disease mechanisms, and prospective intervention strategies. J Clin Invest.

[15] Imad HA, Phadungsombat J, Nakayama EE, Suzuki K, Ibrahim AM, Afaa A (2021). Clinical features of acute chikungunya virus infection in children and adults during an outbreak in the Maldives. Am J Trop Med Hyg.

[16] Ganesan V, Duan B, Reid S (2017). Chikungunya virus: pathophysiology, mechanism, and modeling. Viruses.

[17] Silva LCM, Platner FS, Fonseca LS, Rossato VF, Andrade DCP, Valente JS (2022). Ocular manifestations of chikungunya infection: a systematic review. Pathogens.

[18] Traverse EM, Millsapps EM, Underwood EC, Hopkins HK, Young M, Barr KL (2022). Chikungunya immunopathology as it presents in different organ systems. Viruses.

[19] Vu DM, Jungkind D, LaBeaud AD (2017). Chikungunya virus. Clin Lab Med.

[20] Aurore AC, Couderc T, Dueymes JM, Deligny C, Lecuit M, Molinié V (2021). The Clinicopathological spectrum of kidney lesions in chikungunya fever: a report of 5 cases with kidney biopsy. Am J Kidney Dis.

[21] Nascimento Costa DM, Machado CE, Neves PD, Brito DJ, Oi S, Barros FH (2022). Chikungunya virus as a trigger for different renal disorders: an exploratory study. J Nephrol.

[22] Mercado M, Acosta-Reyes J, Parra E, Guzmán L, Beltrán M, Gasque P (2018). Renal involvement in fatal cases of Chikungunya virus infection. J Clin Virol.

[23] Costa DMN, Gouveia PAC, Silva GEB, Neves PDMM, Vajgel G, Cavalcante MAGM (2023). The relationship between chikungunya virus and the kidneys: a scoping review. Rev Med Virol.

[24] Economopoulou A, Dominguez M, Helynck B, Sissoko D, Wichmann O, Quenel P (2009). Atypical Chikungunya virus infections: clinical manifestations, mortality and risk factors for severe disease during the 2005-2006 outbreak on Réunion. Epidemiol Infect.

[25] Hamid A, Dhrolia MF, Qureshi R, Imtiaz S, Ahmad A (2018). Acute kidney injury secondary to rhabdomyolysis: a rare presentation of Chikungunya fever. J Coll Physicians Surg Pak.

[26] Sharp TM, Keating MK, Shieh WJ, Bhatnagar J, Bollweg BC, Levine R (2021). Clinical characteristics, histopathology, and tissue immunolocalization of Chikungunya virus antigen in fatal cases. Clin Infect Dis.

[27] Aurore AC, Couderc T, Dueymes JM, Deligny C, Lecuit M, Molinié V (2021). The Clinicopathological spectrum of kidney lesions in Chikungunya fever: a report of 5 cases with kidney biopsy. Am J Kidney Dis.

[28] Coelho JL, Israel KCP, Machado CEE, Muniz MPR, Gatto GC, Barros FHS (2021). Thrombotic microangiopathy associated with arboviral infection: report of 3 cases. PLoS Negl Trop Dis.

[29] Solanki BS, Arya SC, Maheshwari P (2007). Chikungunya disease with nephritic presentation. Int J Clin Pract.

[30] Cai L, Hu X, Liu S, Wang L, Lu H, Tu H (2023). The research progress of Chikungunya fever. Front Public Health.

[31] Oliveira JL, Nogueira IA, Amaral JK, Campos LR, Mendonça MMM, Ricarte MB (2023). Extra-articular manifestations of Chikungunya. Rev Soc Bras Med Trop.

[32] Musso D, Gubler DJ (2016). Zika virus. Clin Microbiol Rev.

[33] Ferraris P, Yssel H, Missé D (2019). Zika virus infection: an update. Microbes Infect.

[34] Song BH, Yun SI, Woolley M, Lee YM (2017). Zika virus: history, epidemiology, transmission, and clinical presentation. J Neuroimmunol.

[35] Bhardwaj U, Pandey N, Rastogi M, Singh SK (2021). Gist of Zika Virus pathogenesis. Virology.

[36] Cao-Lormeau VM, Blake A, Mons S, Lastère S, Roche C, Vanhomwegen J (2016). Guillain-Barré Syndrome outbreak associated with Zika virus infection in French Polynesia: a case-control study. Lancet.

[37] Mlakar J, Korva M, Tul N, Popovic M, Poljsak-Prijatelj M, Mraz J (2016). Zika virus associated with microcephaly. N Engl J Med.

[38] Giraldo MI, Gonzalez-Orozco M, Rajsbaum R (2023). Pathogenesis of Zika virus infection. Annu Rev Pathol.

[39] Brasil P, Pereira JP, Moreira ME, Nogueira RMR, Damasceno L, Wakimoto M (2016). Zika virus infection in pregnant women in Rio de Janeiro. N Engl J Med.

[40] Reslan A, Haddad JG, Koundi LM, Desprès P, Bascands JL, Gadea G (2021). Zika virus growth in human kidney cells is restricted by an elevated glucose level. Int J Mol Sci.

[41] Alcendor DJ (2018). Zika virus infection and implications for kidney disease. J Mol Med.

[42] Burdmann EA (2019). Flaviviruses and kidney diseases. Adv Chronic Kidney Dis.

[43] Terzian ACB, Zini N, Sacchetto L, Rocha RF, Parra MCP, Del Sarto JL (2018). Evidence of natural Zika virus infection in neotropical non-human primates in Brazil. Sci Rep.

[44] Tiwari SK, Dang J, Qin Y, Lichinchi G, Bansal V, Rana TM (2017). Zika virus infection reprograms global transcription of host cells to allow sustained infection. Emerg Microbes Infect.

[45] Chen J, Yang YF, Chen J, Zhou X, Dong Z, Chen T (2017). Zika virus infects renal proximal tubular epithelial cells with prolonged persistency and cytopathic effects. Emerg Microbes Infect.

[46] Martines RB, Bhatnagar J, Ramos AMO, Davi HPF, Iglezias SDA, Kanamura CT (2016). Pathology of congenital Zika syndrome in Brazil: a case series. Lancet.

[47] Liu T, Tang L, Tang H, Pu J, Gong S, Fang D (2019). Zika virus infection induces acute kidney injury through activating NLRP3 Inflammasome via suppressing Bcl-2. Front Immunol.

[48] Guzman MG, Harris E (2015). Dengue. Lancet.

[49] Salles TS, Sá-Guimarães TE, Alvarenga ESL, Guimarães-Ribeiro V, Meneses MDF, Castro-Salles PF (2018). History, epidemiology and diagnostics of dengue in the American and Brazilian contexts: a review. Parasit Vectors.

[50] Kularatne SA, Dalugama C (2022). Dengue infection: global importance, immunopathology and management. Clin Med.

[51] Srisawat N, Thisyakorn U, Ismail Z, Rafiq K, Gubler DJ (2022). World dengue day: a call for action. PLoS Negl Trop Dis.

[52] Srisawat N, Gubler DJ, Pangestu T, Thisyakorn U, Ismail Z, Goh D (2023). Proceedings of the 5^th^ Asia Dengue Summit. Trop Med Infect Dis.

[53] World Health Organization (2009). Dengue guidelines for diagnosis, treatment, prevention and control.

[54] Oliveira LLS, Alves FAV, Rabelo K, Moragas LJ, Mohana-Borges R, Carvalho JJ (2022). Immunopathology of Renal Tissue in Fatal Cases of Dengue in Children. Pathogens.

[55] Pagliari C, Quaresma JAS, Kanashiro-Galo L, Carvalho LV, Vitoria WO, Silva WLF (2016). Human kidney damage in fatal dengue hemorrhagic fever results of glomeruli injury mainly induced by IL17. J Clin Virol.

[56] Boonpucknavig V, Bhamarapravati N, Boonpucknavig S, Futrakul P, Tanpaichitr P (1976). Glomerular changes in dengue hemorrhagic fever. Arch Pathol Lab Med.

[57] Lizarraga KJ, Nayer A (2014). Dengue-associated kidney disease. J Nephropathol.

[58] Moorchung N, Jois D, Gupta S, Mutreja D, Patil S (2018). Autopsy in dengue encephalitis: an analysis of three cases. Neurol India.

[59] Upadhaya BK, Sharma A, Khaira A, Dinda AK, Agarwal SK, Tiwari SC (2010). Transient IgA nephropathy with acute kidney injury in a patient with dengue fever. Saudi J Kidney Dis Transpl.

[60] Diptyanusa A, Phumratanaprapin W, Phonrat B, Poovorawan K, Hanboonkunupakarn B, Sriboonvorakul N (2019). Characteristics and associated factors of acute kidney injury among adult dengue patients: a retrospective single-center study. PLoS One.

[61] Vasanwala FF, Puvanendran R, Ng JM, Suhail SM (2009). Two cases of self-limiting nephropathies secondary to dengue haemorrhagic fever. Singapore Med J.

[62] Shih HI, Chi CY, Tsai PF, Wang YP, Chien YW (2023). Re-examination of the risk of autoimmune diseases after dengue virus infection: A population-based cohort study. PLoS Negl Trop Dis.

[63] Limothai U, Jantarangsi N, Suphavejkornkij N, Tachaboon S, Dinhuzen J, Chaisuriyong W (2022). Discovery and validation of circulating miRNAs for the clinical prognosis of severe dengue. PLoS Negl Trop Dis.

[64] Bongsebandhu-Phubhakdi C, Supornsilchai V, Aroonparkmongkol S, Limothai U, Tachaboon S, Dinhuzen J (2023). Serum cortisol as a biomarker of severe dengue. Trop Med Infect Dis.

[65] Kusirisin P, Silva GB, Sitprija V, Srisawat N (2023). Acute kidney injury in the tropics. Nephrology (Carlton).

[66] World Health Organization South-East Asia (2011). Comprehensive guidelines for prevention and control of dengue and dengue haemorrhagic fever, revised and expanded edition.

[67] Jasamai M, Yap WB, Sakulpanich A, Jaleel A (2019). Current prevention and potential treatment options for dengue infection. J Pharm Pharm Sci.

[68] Chawla P, Yadav A, Chawla V (2014). Clinical implications and treatment of dengue. Asian Pac J Trop Med.

[69] Bignardi PR, Pinto GR, Boscarioli MLN, Lima RAA, Delfino VDA (2022). Acute kidney injury associated with dengue virus infection: a review. J Bras Nefrol.

[70] Moura No JA, Silva CAB, Moura AF, Suassuna JHR (2019). Emergent arboviruses and renal transplantation: a global challenge. Kidney Int Rep.

[71] Girão ES, Santos BGR, Amaral ES, Costa PEG, Pereira KB, Araújo Fo AH (2017). Chikungunya infection in solid organ transplant recipients. Transplant Proc.

[72] Foresto RD, Santos DWCL, Hazin MAA, Leyton ATZ, Tenório NC, Viana LA (2019). Chikungunya in a kidney transplant recipient: a case report. J Bras Nefrol.

[73] Maia SHF, Brasil IRC, Esmeraldo RM, Ponte CN, Costa RCS, Lira RA (2015). Severe dengue in the early postoperative period after kidney transplantation: two case reports from Hospital Geral de Fortaleza. Rev Soc Bras Med Trop.

[74] Azevedo LS, Carvalho DBM, Matuck T, Alvarenga MF, Morgado L, Magalhães I (2007). Dengue in renal transplant patients: a retrospective analysis. Transplantation.

[75] Weerakkody RM, Patrick JA, Sheriff MHR (2017). Dengue fever in renal transplant patients: a systematic review of literature. BMC Nephrol.

