# Supplementary material for: Common arboviruses and the kidney: a review
Source: J Bras Nefrol. 2024 Jul 29;46(3):e20230168. doi: 10.1590/2175-8239-JBN-2023-0168en (PMC11287847; doi:10.1590/2175-8239-JBN-2023-0168en)
Supplement: Translation

# Translation: Arboviroses comuns e o rim: uma revisão

**DOI:** 10.1590/2175-8239-JBN-2023-0168pt

**Authors:** Gabriel Rotsen Fortes Aguiar, Geraldo Bezerra da Silva, Janaína de Almeida Mota Ramalho, Nattachai Srisawat, Elizabeth de Francesco Daher

**Affiliations:** 1 Universidade Federal do Ceará, Faculdade de Medicina, Programa de Pós-Graduação em Ciências Médicas, Departamento de Medicina Interna, Fortaleza, CE, Brasil.; 2 Universidade de Fortaleza, Centro de Ciências da Saúde, Curso de Medicina, Fortaleza, CE, Brasil.; 3 Chulalongkorn University, Faculty of Medicine, Department of Medicine, Division of Nephrology, Center of Excellence for Critical Care Nephrology, and Tropical Medicine Cluster, Bangkok, Tailândia.

**Keywords:** Arbovírus, Dengue, Vírus Chikungunya, Zika Vírus, Rim, Prognóstico

## Abstract

As arboviroses são endêmicas em vários países e representam um preocupante problema de saúde pública. A mais importante delas é a dengue, que teve um aumento progressivo do número de casos, atingindo a marca dos milhões de casos anuais no Brasil desde a última década. Outras arboviroses de importância para a saúde pública incluem a chikungunya e a Zika, ambas causas de epidemias recentes, e a Febre Amarela, que também tem causado surtos epidêmicos em nosso país. Assim como a maioria das doenças infecciosas, as arboviroses têm o potencial de acometer os rins, por meio de diversos mecanismos que levam à injúria renal, incluindo ação direta dos vírus, inflamação sistêmica, fenômenos hemorrágicos e outras complicações, além da toxicidade de drogas utilizadas no tratamento. Neste artigo de revisão, serão abordados os aspectos epidemiológicos das principais arboviroses no Brasil e em outros países onde estas doenças são endêmicas, os aspectos clínicos e as principais alterações laboratoriais encontradas, incluindo as alterações na função renal. Como as arboviroses se comportam nos pacientes transplantados renais também será descrito. Serão também descritos os mecanismos fisiopatológicos da injúria renal associada às arboviroses e, por fim, o tratamento recomendado para cada doença e as recomendações para o suporte renal neste contexto.

## Chikungunya

O Chikungunya (CHIKV) é um *Alphavirus* da família *Togaviridae* que é transmitido por mosquitos (*Aedes aegypti* e *Aedes albopictus*). “Chikungunya” significa “aqueles que se curvam” e é uma expressão africana que se refere ao movimento que os pacientes com dor nas articulações fazem. A doença pode ser leve e não causar complicações, mas o principal problema é o desenvolvimento de artrite crônica, que pode durar vários meses ou mesmo anos^1–3^.

## Histórico e Epidemiologia

A Chikungunya causa um número significativo de casos a cada ano e surtos frequentes mundialmente^2^. O primeiro relato foi em 1952 e, desde então, mais de cem países registraram infecção por CHIKV, da África à Ásia, da Oceania às Américas e, recentemente, a Europa também foi afetada por arboviroses. Em 2004, o vírus foi detectado no Quênia e em seguida se espalhou para as ilhas do Oceano Índico, onde causou uma grave epidemia^4^.

Uma etapa importante no estabelecimento da transmissão foi o fato de o vírus ter alcançado o ciclo urbano por meio dos dois mosquitos antropofílicos do gênero Aedes, após milhões de casos serem relatados em todo o mundo, até se adaptar totalmente a esse novo ambiente e agora ser uma ameaça real^7^.

O primeiro relato de chikungunya nas Américas foi em 2013, durante o surto em Saint Martin, no Caribe. O fato foi seguido por uma epidemia na América do Sul, que afetou mais de 50 países e, posteriormente, causou cerca de 1 milhão de infecções^8^.

O chikungunya foi detectado pela primeira vez no Brasil em 2014, nos estados da Bahia e do Amapá. Um estudo epidemiológico indicou uma taxa de incidência, taxa de mortalidade e taxa de letalidade de 114,70 e 87,59 por 100.000 habitantes, 0,15 e 0,12 por 100.000, e 0,13% e 0,14% para 2016 e 2017, respectivamente. A maioria dos casos afetou mulheres jovens com idade média entre 20 e 49 anos, pessoas de pele parda e residentes de áreas urbanas^8^.

A adaptação a novos vetores e sua disseminação para climas temperados, associada à expansão urbana desorganizada e à elevada densidade humana, aumentou a incidência da infecção por Chikungunya.

Dessa forma, surgiram preocupações em relação à ocorrência de chikungunya em países de alta renda, onde foram relatados surtos na Itália e na França na última década^5^. Diversos estudos de modelagem preveem que as mudanças climáticas podem causar exposição a arboviroses à medida que os vetores se espalham para outras regiões fora da zona tropical do globo, e que isso também pode levar a um elevado ônus econômico, uma vez que a chikungunya pode causar doenças debilitantes^9-11^.

## Manifestações Clínicas

O CHIKV possui tropismo por diferentes tipos de células humanas. O vírus se replica em até 8 horas e circula nos vasos linfáticos e na corrente sanguínea, com a possibilidade de atingir diferentes locais de infecção. As articulações e músculos são alvos importantes do vírus chikungunya em humanos. Ele causa uma reação inflamatória grave que afeta vários órgãos, com complicações graves no sistema nervoso central e periférico, coração, pulmões, fígado, sistema vascular e rins^12,13^.

A infecção é caracterizada por uma fase aguda, pós-aguda e crônica. A apresentação clínica pode ser leve, com poucos sintomas e sem complicações, ou pode evoluir com manifestações graves, incluindo síndromes neurológicas e doença articular crônica^14^.

### Doença Aguda

A fase aguda dura aproximadamente uma semana. O período de incubação dura de 3 a 7 dias. Em geral, é seguido por febre, calafrios, exantema maculopapular, que geralmente aparece após 3 a 4 dias de infecção, e dor nas articulações. Ao mesmo tempo em que ocorre o pico de viremia, a febre é elevada (>39°C) e persiste por mais de uma semana. A defervescência pode ocorrer após 4 a 5 dias^1,6,15,16^.

A dor articular debilitante começa de 2 a 5 dias após a febre e se manifesta como artrite ou poliartralgia, geralmente bilateral e simétrica, afetando preferencialmente as mãos, pulsos, ombros, joelhos, tornozelos e pés. A maioria dos pacientes se recupera, mas pode ocorrer dor articular crônica, levando à incapacidade grave^6^.

Embora raras, as manifestações neurológicas, que variam de encefalopatia a encefalomielite disseminada aguda, são sintomas preocupantes que podem ocorrer na fase aguda. A síndrome de Guillain-Barré foi descrita no surto de 2006 na Ilha da Reunião^17,18^.

Uma infecção por chikungunya geralmente apresenta alta viremia, que é maior do que a observada em outras arboviroses. A trombocitopenia é menos acentuada do que na dengue e as complicações hemorrágicas são raras. A linfopenia é o achado laboratorial mais comum, com contagens de linfócitos abaixo de 1.000 células/mm^3^. Pode ocorrer um leve aumento das enzimas hepáticas. Foi relatada hipocalcemia, cujos mecanismos não são conhecidos com precisão^1,6,16^.

### Fase Pós-Aguda e Crônica

A fase crônica pode durar vários meses e até anos^6^. A forma crônica da infecção por chikungunya é considerada quando as manifestações clínicas persistem por mais de 3 meses. Idade >40 anos, sexo feminino e aumento da carga viral durante a fase aguda são considerados fatores de risco para doença articular crônica na chikungunya^6,19^.

Existem evidências de um efeito viral direto na doença articular associada ao chikungunya, uma vez que material genético viral é encontrado em biópsias sinoviais, e macrófagos sinoviais são potenciais reservatórios para o CHIKV^14^.

A fisiopatologia da infecção crônica por chikungunya não é totalmente compreendida. Acredita-se que os fenômenos imunológicos sejam mais relevantes do que os efeitos diretos do vírus, que ocorrem em pacientes com CHIKV na forma de uma resposta imunológica anormal. Aqueles com doença articular crônica apresentam níveis mais elevados de IL-6, bem como de IL-7 e uma relação anormal de RANKL para osteoprotegerina, que está associada à doença óssea e, portanto, contribui para a doença articular observada na chikungunya^6^.

## Envolvimento Renal

As complicações renais na chikungunya variam e precisam ser melhor investigadas.

### Patologia

O achado histopatológico mais comum na injúria renal associada ao chikungunya é a nefrite intersticial aguda associada à necrose tubular aguda, infiltrado mononuclear, congestão glomerular e nefroesclerose. Na fase aguda da doença, antígenos virais foram detectados por imunofluorescência no tecido renal, especificamente em células tubulares, mas não há evidência de persistência na fase crônica. Em um estudo de cinco casos com lesões renais associadas ao CHIKV, dois tipos predominantes foram encontrados nas biópsias renais: nefrite intersticial aguda e injúria tubular aguda^20^.

Um estudo no Brasil, que analisou pacientes com injúria renal comprovada por biópsia, estabeleceu que os diagnósticos mais frequentes foram glomeruloesclerose segmentar focal e a maioria estava associada a fenômenos autoimunes, sugerindo que a chikungunya é um gatilho para autoimunidade^21^.

Um estudo que analisou 13 casos fatais de chikungunya na Colômbia constatou que 11 casos apresentaram nefrite intersticial aguda, 10 casos apresentaram congestão e edema glomerular, 5 casos apresentaram necrose tubular aguda, 5 casos apresentaram nefroesclerose e 1 caso apresentou glomerulonefrite membranoproliferativa^22^.

### Doença Renal na Chikungunya

A doença renal na chikungunya varia de 21 a 45%^23^. Os casos graves de chikungunya podem levar à injúria renal aguda (IRA) em uma frequência de até 79% e estão associados a uma maior mortalidade^23^. Um relato de caso descreveu um paciente do sexo masculino com febre, mialgia e anúria^25^. Na investigação, foi detectada IRA e suspeitou-se de rabdomiólise como causa, induzida por chikungunya^25^.

Um estudo que analisou óbitos ocorridos em Porto Rico em 2014 identificou trinta casos fatais infectados por CHIKV. Entre os achados histopatológicos das 11 amostras nas quais o antígeno viral foi detectado nos rins, o estudo descreveu glomeruloesclerose extensa em 3 casos, glomeruloesclerose leve/moderada em 5 casos, aterosclerose em 7 casos, infiltrado intersticial de células mononucleares em 10 casos e fibrose intersticial em 7 casos. O CHIKV foi detectado nos glomérulos em 4 pacientes, no tecido conjuntivo intersticial/cápsula em 2 pacientes e no epitélio tubular em um paciente^26^. Outros estudos em que amostras renais de pacientes com CHIKV foram examinadas demonstraram nefrite intersticial e injúria tubular com células epitelioides não necrotizantes, granulomas de células gigantes, glomeruloesclerose segmentar focal e microangiopatia trombótica^27,28^.

Os mecanismos imunológicos estão envolvidos no processo da doença renal por CHIKV. Existe um desequilíbrio na resposta imunológica com evidências de produção de anticorpos do tipo IgM e formação de autoanticorpos, incluindo crioglobulinas, que possuem propriedades prejudiciais aos rins^23^. O vírus pode escapar do sistema imunológico e também pode haver uma interação entre os genes e o ambiente que determina a resposta de cada paciente à infecção^23^. O tecido renal também pode servir como reservatório do CHIKV, já que esse vírus tem a capacidade de persistir em vários órgãos e tecidos^23^.

Há evidências de que o chikungunya é um fator desencadeante de várias doenças renais, incluindo a glomerulonefrite^23^. Em um estudo com 15 pacientes com chikungunya no Brasil submetidos à biópsia renal foram identificadas as seguintes lesões histopatológicas: glomeruloesclerose segmentar focal (GESF), nefrite lúpica classe IV, glomerulonefrite crescêntica, microangiopatia trombótica, vasculite pauci-imune, nefropatia membranosa PLA2R positiva e glomeruloesclerose colapsante^23^.

A fisiopatologia do envolvimento renal na infecção pelo vírus chikungunya é mostrada na Figura 1.

**Figura 1:** Fisiopatologia do envolvimento renal na infecção pelo vírus Chikungunya. MAT: Microangiopatia Trombótica; NIA: Nefrite Intersticial Aguda.

### Achados Laboratoriais

Foram encontradas proteinúria e hematúria no CHIKV e são mais frequentes em indivíduos com artrite^23^. A proteinúria é encontrada em 10 a 20% dos casos e a hematúria em menos de 5% dos casos. O aumento da creatinina, que classifica os pacientes com IRA ou doença renal crônica (DRC), é uma complicação relativamente frequente do CHIKV e é descrita em 5,2% a 79% dos casos^23^.

### Outras Manifestações Renais

A síndrome nefrítica foi descrita em associação com a chikungunya em um jovem da Índia que não apresentava histórico de doença renal. O material genético do CHIKV foi detectado no soro do paciente^29^.

## Tratamento

O tratamento da chikungunya é essencialmente para o alívio da dor na ausência de uma droga específica^31^, e o tratamento permanece sintomático. Para algumas glomerulonefrites específicas desencadeadas pelo chikungunya, como a GESF ou a microangiopatia trombótica, existem tratamentos específicos com corticosteroides, imunossupressores, plasmaférese e outros. No entanto, não há evidências suficientes para nenhum desses tratamentos no chikungunya^31^.

## Zika

O vírus Zika (ZIKV) é um *Flavivirus* da família *Flaviviridae* que foi identificado pela primeira vez em 1947 na região da floresta de mesmo nome, em Uganda. A primeira infecção humana foi descrita alguns anos depois na Nigéria e, por muitos anos, apenas alguns casos foram documentados^32^. A transmissão também ocorre por meio da picada do mosquito Aedes e, além da transmissão perinatal, sexual e pelo leite materno, foi descrita a transmissão entre humanos.

## Histórico e Epidemiologia

Até 2007, o Zika foi responsável somente por pequenos surtos, quando uma grande epidemia ocorreu na ilha de Yap, no Pacífico, afetando cerca de 75% da população. Os médicos locais descreveram inicialmente uma “doença semelhante à dengue”, mas alguns pacientes, que relataram apenas febre subjetiva e conjuntivite, levantaram suspeitas. Essa epidemia foi seguida por uma epidemia maior na Polinésia Francesa em 2013-2014, com mais de 30.000 casos^33^. Em 2015, o ZIKV foi detectado nas Américas. Desde então, 33 países relataram transmissão autóctone e um aumento significativo em algumas complicações, incluindo microcefalia e síndrome de Guillain-Barré^33^. O ZIKV também foi detectado na Europa, o que sugere uma rápida disseminação para outras partes do mundo, semelhante ao chikungunya e à dengue^32,33^.

## Manifestações Clínicas

O ZIKV tem um período de incubação de 3 a 14 dias. Os sintomas iniciais são semelhantes aos da influenza, com febre e mal-estar. O Zika é caracterizado por exantema maculopapular com coceira e dor articular. Embora raro, o ZIKV pode causar a síndrome de Guillain-Barré e outras complicações neurológicas em adultos^33–35^.

Surtos recentes levantaram questões preocupantes sobre a possível gravidade do ZIKV, principalmente em relação à sua associação com complicações neonatais^38^. No Brasil, um estudo com gestantes infectadas pelo ZIKV mostrou que 42% dos fetos apresentavam anormalidades ultrassonográficas^39^. A doença congênita causada pelo Zika tem uma apresentação variável, que vai desde o óbito fetal até a microcefalia e outras manifestações^33^.

A ocorrência do ZIKV tornou-se uma preocupação de saúde pública devido ao seu impacto na saúde neonatal e nas complicações neurológicas, especialmente a síndrome de Guillain-Barré em adultos, durante os surtos^32^. Acredita-se que a neuropatia associada ao Zika seja causada por anticorpos produzidos contra o ZIKV que reagem aos nervos periféricos e outras estruturas^33^.

## Envolvimento Renal

O envolvimento renal no Zika não é bem estudado. Existem algumas evidências de possíveis mecanismos que causam doença renal associada ao Zika, incluindo excreção persistente do ZIKV na urina, alta suscetibilidade das células glomerulares e tubulares à infecção por ZIKV e liberação de citocinas associadas à inflamação^40^. O ZIKV é capaz de invadir e se replicar em células glomerulares, e a suscetibilidade dessas células ao vírus contribui para sua persistência na urina de pacientes infectados^43^.

Um estudo experimental com diferentes tipos de células (microglia, fibroblastos, rim embrionário e macrófagos) inoculadas com ZIKV indicou que a linhagem celular de rim embrionário humano é um ambiente adequado para a replicação do ZIKV^44^. Um estudo recente investigou se a infecção pelo ZIKV em células renais é dependente dos níveis de glicose e demonstrou que os níveis de glicose influenciam a replicação do ZIKV e têm impacto na sobrevida das células renais^40^. Estudos experimentais também demonstraram que o RNA do ZIKV foi detectado tanto em células glomerulares quanto tubulares. Verificou-se um aumento de células CD8+ nos rins infectados pelo ZIKV^45^. Em uma série de casos de 5 bebês que faleceram de infecção por ZIKV, não foram encontrados antígenos virais nos rins^46^.

Sabe-se, a partir de modelos experimentais de infecção pelo vírus Zika, que os rins são danificados, com lesão tubular significativa, fibrose tubulointersticial (14 dias após a infecção) e infiltração de células imunológicas^47^. Nesse modelo animal de IRA associada ao Zika, foram detectados biomarcadores não tradicionais de injúria renal, como a molécula de injúria renal-1 (KIM-1) e a lipocalina associada à gelatinase neutrofílica (NGAL)^47^. Existem evidências de que o vírus Zika induz inflamação e injúria renal por meio de mecanismos que envolvem o inflamassoma NLRP3 (*Nod-like receptor 3*) e a secreção de interleucina-1β (IL-1β)^47^.

A Figura 2 mostra uma ilustração do envolvimento renal associado ao Zika.

**Figura 2:** Fisiopatologia do envolvimento renal na infecção pelo vírus Zika.

## Tratamento

Da mesma forma que em outras arboviroses, não há tratamento específico para o Zika, nenhum antiviral ou outro medicamento que seja eficaz. O tratamento é limitado ao alívio dos sintomas^33^. Não há recomendações específicas de tratamento para a IRA induzida por Zika. Devemos seguir as diretrizes atuais para o tratamento da IRA.

## Dengue

A dengue é um vírus *Flaviviridae* com 4 sorotipos (DENV 1-4) transmitidos por mosquitos *Aedes* ^48^. A doença pode se manifestar desde uma infecção assintomática até uma doença grave com falência de múltiplos órgãos. Atualmente, a dengue é um grande problema de saúde pública devido à sua ampla distribuição no mundo e ao grande número de pessoas expostas ao risco de infecção^48^.

## Histórico e Epidemiologia

Os primeiros registros de dengue remontam à Ásia em tempos antigos e, por volta do século XVIII, várias epidemias de doença semelhante à dengue haviam sido registradas na Ásia e nas Américas^49^. Nos séculos XIX e XX, o vírus possivelmente se espalhou pelo mundo tropical^49^.

A dengue é hoje considerada uma doença hiperendêmica em muitas partes do mundo, com casos relatados em mais de 100 países. Nos últimos 50 anos, o número de casos aumentou 30 vezes^48,50^. A Organização Mundial da Saúde (OMS) tem como objetivo reduzir a zero o número de óbitos evitáveis por dengue até 2030. Para atingir esse objetivo, devem ser tomadas medidas conjuntas^51,52^.

## Manifestações Clínicas

A maioria dos casos de dengue é assintomática. Após um período de incubação de cerca de 4 a 8 dias, os sintomas aparecem repentinamente, incluindo febre, cefaleia, dor retro-orbital, mialgia e outros sintomas menos específicos. Na maioria dos casos, a recuperação ocorre dentro de alguns dias, e uma pequena proporção de pacientes evolui com complicações, principalmente devido ao extravasamento plasmático, com ou sem manifestações hemorrágicas^48,49^. A doença pode passar por três fases: febre, fase crítica e recuperação. Na fase aguda, as manifestações se assemelham às de outras doenças virais, podendo essa fase durar de 3 a 7 dias. Os exames laboratoriais podem mostrar leucopenia, trombocitopenia e hemoconcentração associadas a um aumento das enzimas hepáticas^48,49^.

Existe uma fase crítica que coincide com o declínio da febre, na qual a permeabilidade capilar aumenta e pode levar à hipovolemia e à falência de órgãos. Acredita-se que uma resposta imune anormal cause uma tempestade de citocinas com a liberação de diversos fatores solúveis, incluindo TNF-α, IL-6, IL-8, IL-10 e IL-12, levando a um aumento da permeabilidade vascular e extravasamento plasmático. Durante a fase de recuperação, esse vazamento vascular deve terminar com a reabsorção de fluidos^48,50^.

Em 2009, a OMS propôs uma nova classificação para a dengue com base na presença de sinais de alerta, substituindo a antiga classificação de dengue clássica x dengue hemorrágica^48,50,53^.

## Envolvimento Renal

### Patologia

Estudos de casos em adultos e crianças com dengue que foram submetidos a biópsias renais revelaram os seguintes achados: congestão glomerular, infiltrado de células plasmáticas, congestão capilar glomerular e peritubular, cilindros hialinos, edema endotelial e proliferação mesangial^54,55^.

Em pacientes infectados com dengue, depósitos de IgG, IgM e C3 são encontrados nos glomérulos em aproximadamente 50% dos casos^57^.

O envolvimento do sistema tubular também foi investigado, com desnudamento do epitélio tubular, células tubulares com núcleos picnóticos, atrofia/fibrose tubular focal e nefrite tubulointersticial^54,55^.

Em uma série de casos da Índia, foram analisadas as biópsias de três pacientes infectados com dengue e observou-se necrose tubular proximal com hemorragia no espaço de Bowman em todos os casos e cilindros eritrocitários em dois casos^58^.

Depósitos de IgA foram detectados em um relato de caso, sugerindo nefropatia por IgA, mas essa associação ainda não está bem explorada^59^.

### Injúria Renal Aguda

O mecanismo da IRA associada à dengue é complexo. A injúria renal tubular pode estar associada tanto a anormalidades hemodinâmicas quanto a efeitos virais diretos. Outros fatores, como lesão induzida por citocinas, hemólise e rabdomiólise, desempenham um papel importante na IRA causada pela dengue^50,60^.

Na Tailândia, propôs-se uma classificação paralela da dengue que categoriza a gravidade da doença em febre da dengue, febre hemorrágica da dengue e síndrome do choque da dengue para capturar a ocorrência de IRA ao longo do desenvolvimento da doença^57^. Os autores analisaram os prontuários médicos de 1.484 pacientes com dengue de um centro de patologia local e encontraram uma prevalência de IRA de 4,8%. Eles concluíram que idade, sexo masculino, diabetes, obesidade, dengue grave e infecção bacteriana secundária foram significativamente associados ao desenvolvimento de IRA^60^.

A fisiopatologia do envolvimento dos rins na dengue é mostrada na Figura 3.

**Figura 3:** Fisiopatologia do envolvimento renal na Dengue. SRIS: Síndrome da Resposta Inflamatória Sistêmica; MAT: Microangiopatia Trombótica; SHU: Síndrome hemolítico-urêmica.

### Achados Laboratoriais

A hematúria foi relatada em até 12,5% dos pacientes com FHD^11^. A proteinúria é observada em mais de 7% dos casos de dengue^61^ relataram dois pacientes com dengue e proteinúria nefrótica^61^. Um estudo realizado na Tailândia demonstrou anormalidades no sedimento urinário em pacientes com dengue e IRA, incluindo proteinúria (70,4%), hematúria (59,3%) e piúria (40,7%). A acidose metabólica também foi encontrada em 24,3% dos pacientes^59^.

### Citocinas

As citocinas mais frequentemente envolvidas são IL-17, IL-18 e TNF-α. Distúrbios autoimunes têm sido associados à dengue, e pacientes com essa infecção são atualmente considerados indivíduos com alto risco de desenvolver diversas doenças, como artrite, esclerose múltipla, vasculite, lúpus e outras doenças autoimunes^62^.

### MicroRNAs

Pequenos RNAs não codificadores, os chamados microRNAs (miRNAs), que têm comprimento de 20 a 22 nucleotídeos, são importantes para o controle pós-transcricional da expressão gênica. Os pacientes com dengue grave apresentam um padrão diferente de expressão de miRNA, e há miRNAs que podem ser usados como marcadores prognósticos para a gravidade da dengue, sendo o miR-574-5p e o miR-1246 os mais promissores^63^.

### Cortisol

Há evidências de alterações do cortisol na dengue. Os pacientes com dengue grave parecem ter níveis aumentados de cortisol, mas o significado exato disso ainda não está claro^64^.

## Tratamento

A terapia de suporte, juntamente com a administração de líquidos, é o principal tratamento para a dengue. Há um debate contínuo sobre o uso de corticosteroides parenterais na dengue grave^65^. Existe uma diretriz da OMS para o manejo da dengue^64^. Os pacientes devem ser advertidos contra o uso de drogas antiinflamatórias devido ao risco de hemorragia na trombocitopenia grave^50,67,68^.

Não existe tratamento específico para a IRA associada à dengue, mas uma etapa crucial para o manejo do paciente é o suporte hídrico, mantendo a perfusão adequada^69^. A ingestão de líquidos deve ser cuidadosamente monitorada para evitar sobrecarga hídrica, uma vez que o extravasamento intravascular na dengue é um risco eminente e piora o prognóstico. Um fator que contribui para a IRA na dengue é a rabdomiólise, embora sua incidência real não seja precisamente conhecida. No entanto, essa é outra razão pela qual a administração de líquidos na dengue é importante e essencial para prevenir a IRA ou, pelo menos, reduzir sua gravidade. Os distúrbios eletrolíticos também são uma preocupação na dengue, e o mais frequente é a hiponatremia. Portanto, a reposição volêmica deve ter maior tonicidade nesses casos^69^.

A IRA grave associada à dengue pode exigir diálise, que é frequentemente indicada quando os pacientes apresentam uremia, hipercatabolismo, acidose metabólica, hipercalemia e hipervolemia refratária às medições clínicas^69^. A dengue pode causar instabilidade hemodinâmica, sendo assim, a hemodiálise contínua tem sido indicada em pacientes com IRA associada à dengue nesse contexto^69^.

## Transplante Renal

As infecções por arbovírus também são uma preocupação para os pacientes de transplante renal. Aspectos específicos da chikungunya, Zika e dengue merecem destaque no contexto do transplante renal.

Há relatos de que a infecção por chikungunya ocorre com mais frequência em pacientes imunocomprometidos^70^. Existem poucos estudos sobre o chikungunya em pacientes transplantados renais sem complicações e com baixa mortalidade^71^. Além disso, durante uma infecção por chikungunya, o enxerto renal pode perder a função, mas recupera-se totalmente após o episódio agudo^71^.

O Zika pode ser transmitido por transfusões e transplantes^70^. Infecções por Zika em pacientes transplantados renais não são relatadas com frequência, mas quando ocorrem, a infecção parece ser pior em receptores de transplante, com perda da função do aloenxerto, porém baixa mortalidade^70^. É preocupante o fato de que o vírus Zika pode persistir nos órgãos mesmo na ausência de viremia, mas não está claro qual é o risco de complicações^70^.

A infecção pelo vírus da dengue pode causar viremia prolongada e doença assintomática, tornando a transmissão por meio de transplante renal um risco real^70^. Apesar desse risco potencial, os testes sorológicos para o vírus da dengue não são realizados rotineiramente em doadores ou receptores renais. A incidência de infecção por dengue não parece ser maior no período pós-transplante. A razão para isso é que a maioria dos pacientes já foi infectada antes do transplante, e o vírus parece ser menos patogênico quando transmitido por transfusão de sangue ou transplante de órgãos^70^.

Quanto às manifestações clínicas, a dengue em pacientes transplantados renais é leve na maioria dos casos^70^. Existem poucos relatos de complicações graves associadas à dengue em receptores de transplante renal, incluindo óbito, e alguns autores recomendam a triagem para dengue no período pré-operatório^73^. Estudos anteriores sobre a dengue associada ao transplante renal mostram um curso clínico não diferente do observado em hospedeiros imunocompetentes, com uma baixa taxa de mortalidade^74^. Alguns sintomas, como febre, mialgia, artralgia e cefaleia, são ainda menos frequentes em pacientes transplantados renais com dengue do que na população em geral, mas uma taxa de mortalidade mais elevada foi observada em algumas séries (8,9% versus 3,7% em pacientes não transplantados)^75^. A imunossupressão está possivelmente associada ao desfecho dos pacientes. Altas doses de corticosteroides (>7,5 mg por dia) estão associadas à doença mais grave. O tacrolimus tem sido associado a um maior risco de sangramento, enquanto a ciclosporina está associada a uma forma mais leve da doença^70^.

## Conclusão

As arboviroses são endêmicas na maioria dos países tropicais e podem causar injúria renal. Existem três arboviroses principais, a saber, chikungunya, Zika e dengue, que apresentam surtos periódicos em muitas partes do mundo. Há diferentes mecanismos pelos quais esses vírus podem causar danos renais, incluindo o efeito direto do vírus. Os receptores de transplante renal também podem ser afetados; embora possam ocorrer danos ao transplante, a função renal geralmente se recupera após o desaparecimento da infecção. Nas áreas endêmicas, deve-se realizar o monitoramento contínuo, juntamente com o monitoramento da função renal em todos os pacientes infectados, incluindo os receptores de transplante renal das áreas endêmicas. Não existe tratamento específico disponível para essas infecções virais e a terapia de suporte, incluindo diálise para injúria renal grave, continua sendo o pilar do tratamento.
